# Direct band gap and anisotropic transport of ZnSb monolayers tuned by hydrogenation and strain†

**DOI:** 10.1039/d1ra08619g

**Published:** 2022-01-20

**Authors:** Zhizi Guan, Wei Yang, Hongfa Wang, Hailong Wang, Junwen Li

**Affiliations:** CAS Key Laboratory of Mechanical Behavior and Design of Materials, Department of Modern Mechanics, CAS Center for Excellence in Complex System Mechanics, University of Science and Technology of China Hefei Anhui 230 027 China hailwang@ustc.edu.cn; DFTWorks LLC Oakton, VA 22 124 USA junwen.li@dftworks.com

## Abstract

Using first-principles density-functional theory simulations, we explore the effects of hydrogenation and strain on the mechanical, electronic and transport properties of two-dimensional ZnSb monolayers. We find that the fully hydrogenated ZnSb monolayer exhibits large mechanical anisotropy between armchair and zigzag directions and the biaxial tensile strain reduces the anisotropy. In addition, we find that the hydrogenation can induce a metal-to-semiconductor transition with a direct band gap of 1.12 (1.92) eV using the PBE (HSE) functional. With biaxial strains, the band gaps decrease monotonically and remain direct for strains smaller than 5%. Moreover, large transport anisotropy is demonstrated by computing the effective masses of charge carriers along the asymmetric armchair and zigzag directions. We further reveal that strain can significantly tune the effective masses and a 3% strain can even switch the effective transport direction for holes. Our simulations suggest that the hydrogenated ZnSb monolayer is a promising candidate for electronic and opto-electronic applications with controllable modification *via* strain engineering.

## Abstract 2

Using first-principles density-functional theory simulations, we explore the effects of hydrogenation and strain on the mechanical, electronic and transport properties of two-dimensional ZnSb monolayers.

## Graphical abstract

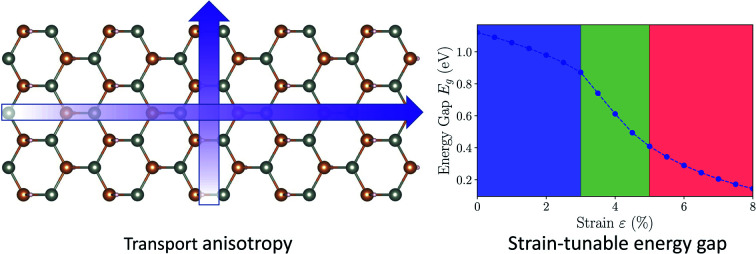

## 1 Introduction

The discovery of graphene in 2004 has triggered rapidly growing interest in exploring novel two-dimensional (2D) ultra-thin materials^1,2^ such as hexagonal boron–nitride (h-BN),^3,4^ transition metal dichalcogenides (TMDCs)^5,6^ and MXenes^7,8^ with diverse physical properties. These emerging 2D materials are mainly synthesized *via* chemical vapor deposition or exfoliated from their mother compounds with inherent 2D layered structures.^9–11^ Recently, ultra-thin zinc antimonide (ZnSb), a new member of the 2D material family, is made available *via* transforming the sp^3^-hybridized three dimensional (3D) crystal ZnSb to a structure configuration with dominating sp^2^ bonding by lithiation.^12^ The intrinsic ZnSb monolayer is found to be metallic while a tunable direct band gap is desirable for a wide range of technological applications.^13^ Therefore, it would be of great significance to identify effective routes of tuning its properties to explore the potential and expand the application of this novel 2D material.

In two-dimensional systems, the surface exposure of a large portion of constituent atoms or even all of them makes surface passivation an effective manner of modifying the properties of a material.^14–17^ In particular, hydrogenation has been demonstrated to have remarkable and diverse impacts. For example, hydrogenating graphene can open up a large gap, which was first computationally studied and then experimentally verified.^18,19^ For TMDCs, hydrogenation can lead to a structural phase transition for the MoTe_2_ monolayer and can saturate the sulfur vacancies in the MoS_2_ monolayer to achieve tunable doping.^20,21^ Crisotomo *et al.*^22^ predict that the hydrogenated TlBi film is topologically non-trivial with a large band gap of 855 meV and, therefore, could be used in room temperature application. Xu *et al.*^23^ present that hydrogenation can stabilize borophene to get borophane possessing a perfect linear band dispersion and Fermi velocities higher than those of graphene. Moreover, nanostructured materials can endure much larger strain compared with their bulk counterparts.^24–26^ And strain engineering has been well utilized to tune the electronic and optical properties, band gaps and charge transport properties.^27–33^ Therefore, it is also critical to characterize and understand its strain response to realize potential applications of the emerging 2D ZnSb.

In this paper, by performing density-functional theory calculations, we study the effect of hydrogenation and biaxial strain on the electronic structures to obtain a tunable direct band gap and the underlying mechanism is understood based on the changes in the energy states near the Fermi level. In addition, we report the anisotropic mechanical and transport properties of fully hydrogenated ZnSb monolayers characterized by the Young's modulus and effective masses of charge carriers, respectively.

This paper is organized as follows: in Section 1, we review the computational approaches employed in studying the structural energetics and electronic structures. In Section 2, we present and discuss the computational results. First, we study the atomic structures and the mechanical properties. Next, we investigate the electronic structures, density of states and near-gap states as well as their strain response. Lastly, we demonstrate the transport anisotropy from the aspects of the band anisotropy and effective masses along different orientations.

## 2 Methods

We carry out the density-functional theory calculations using the Vienna *ab initio* simulation package (VASP), with the exchange–correlation functional described by Perdew–Burke–Ernzerhof (PBE) generalized gradient approximation and the interaction between core and valence electrons by frozen-core projector-augmented wave method.^34–36^ The cutoff energy used in plane wave basis expansion is set to be 500 eV and a vacuum space of 15 Å along the direction normal to the ZnSb sheet is employed to eliminate the interaction between artificial periodic layers. For Brillouin zone sampling, we use the Monkhorst–Pack method of a 15 × 15 × 1 *k* grid. All atoms are allowed to relax until the forces acting on each atom are less than 0.001 eV Å^−1^. We also consider the dipole correction oriented perpendicularly to the 2D surface. It is known that the PBE functional can lead to a band gap underestimation due to the self-interaction error.^37^ Hence, we also perform the electronic structure calculations using the Heyd–Scuseria–Ernzerhof (HSE) hybrid functional.^38,39^

Differential hydrogen absorption energy Δ*E*_H_ is used to describe the energetics of hydrogenated sheets, which is defined asΔ*E*_H_ = *E*_ZnSb+*n*H_ − *E*_ZnSb+(*n*−1)H_ − (1/2)*E*_H_2__,where *E*_ZnSb+*n*H_ and *E*_ZnSb+(*n*−1)H_ are the total energies for the 2D ZnSb system with *n* and *n* − 1 hydrogen atoms absorbed, respectively, and *E*_H_2__ is the energy of a gas phase hydrogen molecule. To study the strain effect, we apply biaxial strains defined as *ε* = (*L* − *L*_0_)/*L*_0_ on the lattice vectors and relax the atomic coordinates for each strain, where *L*_0_ and *L* are the lattice constants before and after applying tensile strains, respectively.

For the smooth plotting of iso-energy contour to show the band anisotropy, a finer *k*-grid is needed and we construct the first-principles tight-binding Hamiltonian to reduce the computational cost. We first use Quantum ESPRESSO to carry out the electronic structure and output the wave functions on a coarse *k* grid (10 × 6 × 1), and then perform the Wannier interpolation technique with Wannier90 code to get the eigen energies on a *k* grid of 200 × 200 × 1.^40,41^

## 3 Results and discussion

### 3.1 Structure models

**Fig. 1:**
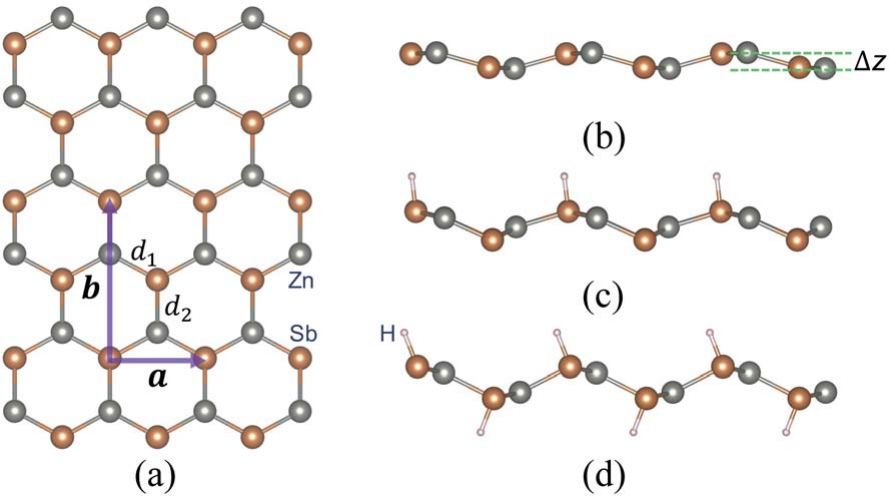
A schematic depicting the crystal structure of a ZnSb monolayer sheet with (a) top view and (b) side view. The unit cell (purple) with lattice vectors **a** and **b** is shown in (a). Two nonequivalent Zn–Sb bonds are labeled with *d*_1_ and *d*_2_. Δ*z* in (b) indicates the buckling of the two-dimensional sheet defined as the distance between two outermost atom layers (Zn or Sb). (c) and (d) demonstrate the side views of ZnSb monolayers with half and full hydrogenation, respectively. The orange and the light gray dots in the crystal structure represent the Sb and Zn atoms, respectively.

We first study the structural properties of the ZnSb monolayer. As shown in Fig. 1, the unit cell is rectangular and the lattice constants of a pristine ZnSb monolayer sheet are calculated to be *a* = 4.52 Å and *b* = 7.45 Å, respectively. Along the direction normal to the sheet plane, we can observe a buckling of Δ*z* = 0.64 Å which is defined as the distance between two outermost layers consisting of Zn or Sb atoms. There are two Sb atoms in an unit cell. The situations with one or two Sb atoms passivated by hydrogen atoms are referred to as half or full hydrogenation, which will induce different changes in the lattice constants. The choice of the location of H on the ZnSb sheet is based on the difference in electronegativities between Zn and Sb atoms that are 1.65 (Zn) and 2.05 (Sb), respectively. This indicates that there would be a charge transfer from Zn to Sb when they form bonds. The Bader charge analysis shows a charge transfer of 0.23*e*^−^, leading to positively charged Zn and negatively charged Sb. H that tends to be positively charged is expected to bond with Sb. We further confirm the choice of absorption position of H by performing the structural relaxation with H initially located close to Zn. The relaxation process drives H to move toward Sb and we still end up with the configuration as shown in Fig. 1. With half hydrogenation, *a* reduces to 3.86 Å while *b* is slightly increased to 7.48 Å. On the contrary, the full hydrogenation increases *a* to 4.60 Å and *b* to 7.55 Å. The ratio *a*/*b* is very close between the pristine (0.61) and fully hydrogenated (0.61) monolayer while the half hydrogenated sheet exhibits the smallest value (0.52). The bucklings are increased to 1.39 Å for half hydrogenation and to 1.21 Å for full hydrogenation. In a pristine sheet, there are two nonequivalent Zn–Sb bonds with bond lengths *d*_1_ = 2.57 Å and *d*_2_ = 2.59 Å. Half hydrogenation leads to increased bonds (*d*_1_ = 2.64 Å and *d*_2_ = 2.66 Å), which are nearly the same with the values (*d*_1_ = 2.65 Å and *d*_2_ = 2.66 Å) in the fully hydrogenated sheet. The thermodynamic energetics of hydrogenated ZnSb sheets are indicated by the negative differential hydrogen absorption energies (−0.65 eV and −0.25 eV). The structural parameters discussed above are listed in Table 1. The effect of hydrogenation on the structure could be understood as follows: the Young's modulus in zigzag and armchair directions are 31.37 N m^−1^ and 1.55 N m^−1^, respectively. When half hydrogenation is introduced and leads to more sp^3^ like bonding for the attached Sb atoms, a change in the lattice constant along the zigzag direction can effectively relax the structure, giving us a smaller lattice constant *a* and larger buckling (1.39 Å). The full hydrogenation leads to competitions between the opposite pulling of two sides by the hydrogenation and between the pulling interaction and the bonding interaction of Zn and Sb atoms, making the lattice constants close to the original values (the pristine ZnSb sheet) as shown in Table 1.

**Table 1:** Structural parameters of two-dimensional ZnSb monolayers with no, half and full hydrogenation. *a*, *b*: lattice constants; Δ*z*: buckling denoted by the distance between two outermost layers consisting of Zn or Sb atoms; *d*_1_, *d*_2_: bond lengths of Zn–Sb; *d*_H–Sb_: bond length of H–Sb; Δ*E*_H_: differential hydrogen absorption energy

Configuration	*a* (Å)	*b* (Å)	*a*/*b*	Δ*z* (Å)	*d*_1_ (Å)	*d*_2_ (Å)	*d*_H–Sb_ (Å)	Δ*E*_H_ (eV)
Pristine	4.52	7.45	0.61	0.64	2.57	2.59	—	—
Half-H	3.86	7.48	0.52	1.39	2.64	2.66	1.72	−0.65
Full-H	4.60	7.55	0.61	1.21	2.65	2.66	1.73	−0.25

### 3.2 Young's modulus

**Fig. 2:**
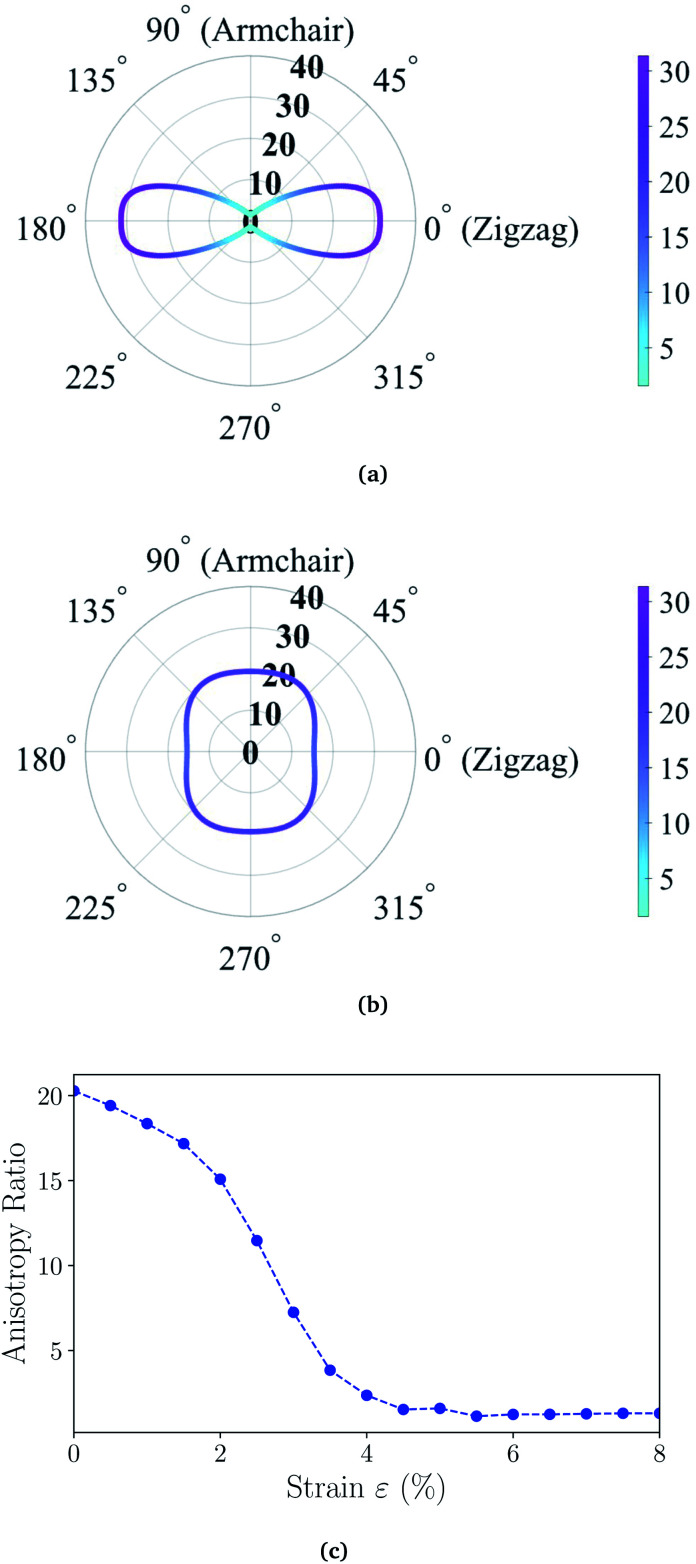
Young's modulus of (a) unstrained ZnSb monolayer sheet and (b) ZnSb monolayer sheet under 8% strain as a function of the in-plane angle *θ* with *θ* = 0 corresponding to the *a* axis (zigzag). (c) The anisotropy ratio as a function of strain, calculated as *Y*_2D_(*θ*)_max_/*Y*_2D_(*θ*)_min_ for each biaxial strain.

To further investigate the mechanical stability, we perform the stiffness matrix analysis for the fully hydrogenated ZnSb monolayer that contains four independent elastic constants: *C*_11_, *C*_22_, *C*_12_ and *C*_66_. It is shown that the Born-Huang stability criteria is met: *C*_11_ (32.212 N m^−1^) > 0, *C*_22_ (1.588 N m^−1^) > 0, *C*_11_ > |*C*_12_| (1.155 N m^−1^), and *C*_66_ (8.743 N m^−1^) > 0.^42,43^ In addition to serving as an indicator of the mechanical stability, these elastic constants are key parameters used to determine the orientation dependence of Young's modulus *Y*_2D_(*θ*) *via* the formula1
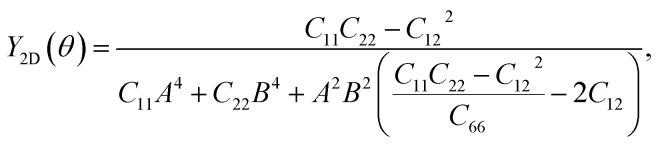
in which *θ* denotes the polar angle relative to the *a* axis (zigzag) in Fig. 1 with *A* and *B* defined as sin *θ* and cos *θ*, respectively. We present the angle-resolved Young's modulus *Y*_2D_(*θ*) for the unstrained ZnSb monolayer in Fig. 2(a). The Young's modulus in zigzag and armchair directions are 31.37 N m^−1^ and 1.55 N m^−1^, respectively, indicating a strong mechanical anisotropy. This can be attributed to the structure anisotropy intrinsic to the zigzag-shaped buckling. In contrast, ZnSb monolayer is more flexible and its stiffness is lower than that of other well studied 2D materials such as graphene and h-BN, whose Young's moduli are 340 N m^−1^ and 271 N m^−1^, respectively.^44,45^

Moreover, the stiffness and mechanical anisotropy can be largely modified by applying biaxial strain as demonstrated in Fig. 2(b) depicting the angle-resolved Young's modulus for ZnSb monolayer under 8% strain. The Young's modulus in zigzag and armchair directions become 15.39 N m^−1^ and 19.41 N m^−1^, respectively. In addition, for the ZnSb monolayers under 3% and 5.5% strains we present their *Y*_2D_(*θ*) in Fig. S1† to demonstrate the evolution of *Y*_2D_(*θ*) as a function of strain. To further quantify the degree of magnitude of mechanical anisotropy, we compute the anisotropy ratio of *Y*_2D_ defined as *Y*_2D_(*θ*)_max_/*Y*_2D_(*θ*)_min_. Starting with 20.24 at 0% strain, the anisotropy ratio is monotonically decreasing with tensile strains and a nearly isotropic state is observed for ZnSb monolayers at a strain of about 5% and larger.

### 3.3 Electronic structures

**Fig. 3:**
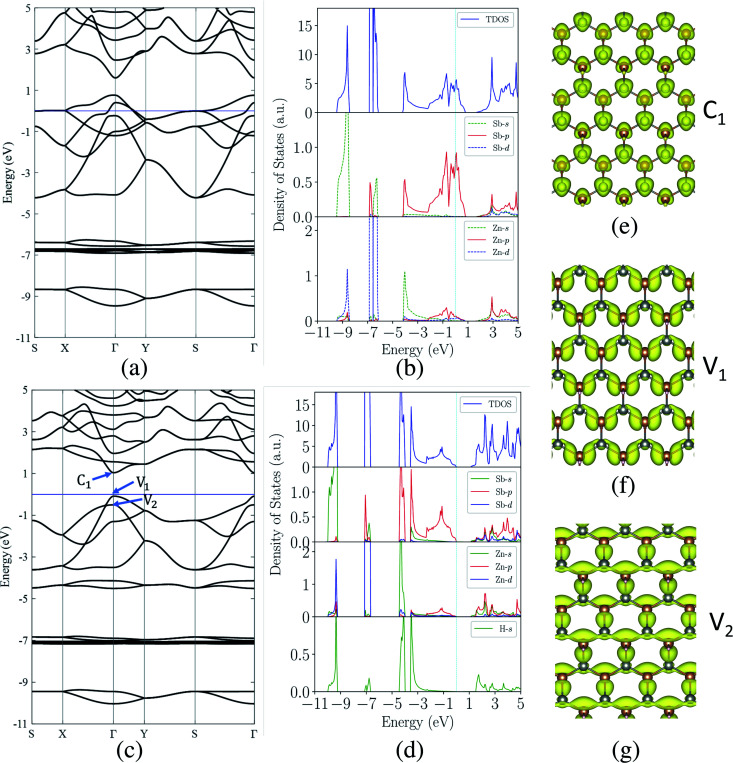
(a) Calculated electronic band structures (PBE) and (b) total and orbital-projected density of states for the pristine ZnSb monolayer. (c) and (d) are for the fully hydrogenated ZnSb monolayer. The Fermi level is set to zero energy. Partial charge density iso-surfaces (e), (f) and (g) correspond to the near-gap energy states labeled with C_1_, V_1_ and V_2_, respectively.

Next, we investigate the electronic properties of the ZnSb monolayers and present the electronic band structure and density of states (DOS) in Fig. 3(a) and (b) for the pristine sheet, respectively. The intense DOS peaks around the Fermi level are dominated by the Sb-p orbitals with marginal contributions from Zn-p orbitals. In Fig. 3(b) and (d) we observe non-zero density of states projected onto Zn p orbital although the valence configuration of a Zn atom is 3d(10)4s(2) with 3p orbitals as the inner shell. When Zn forms bonding with Sb, the surrounding environment of Zn atom is changed, leading to charge redistribution and orbital polarization of valence electrons. This polarization is manifested as the non-zero projection of orbitals onto the p orbital. The contribution from Sb-s orbitals are mainly located in the range of −9.5 eV to −8.5 eV while Zn-d orbitals form the flat bands lying from −7.0 eV to −6.0 eV. The fact that the Sb-p electrons are very active suggests that passivating those orbitals would have significant impact on the electronic structures of ZnSb monolayers.

When one of the two Sb atoms in an unit cell is bonded to hydrogen as shown in Fig. 1(c), the sheet is still metallic while the intensity of DOS around Fermi level is greatly reduced as indicated in Fig. S2† depicting the electronic structure and density of states. With all two Sb atoms passivated by hydrogen as shown in Fig. 1(d), we observe a metal-to-semiconductor transition with a direct band gap of 1.12 eV with both valence band maximum (VBM) and conduction band minimum (CBM) at Γ point as shown in Fig. 3(c). The electronic structure calculation using HSE functional reveals a larger direct band gap of 1.92 eV at Γ point (Fig. S4†). Since the metallicity of pristine monolayer ZnSb is a big obstacle for its semiconducting applications, other methods have been studied to open up a band gap which is either indirect or of small values. Bafekry *et al.* show that the ZnSb bilayers can become semiconducting, but exhibit an indirect gap.^46^ The direct band gaps of fluorinated and chlorinated ZnSb are calculated to be 0.06 (1.0) eV and 0.5 (1.4) eV using PBE (HSE) method, respectively.

**Fig. 4:**
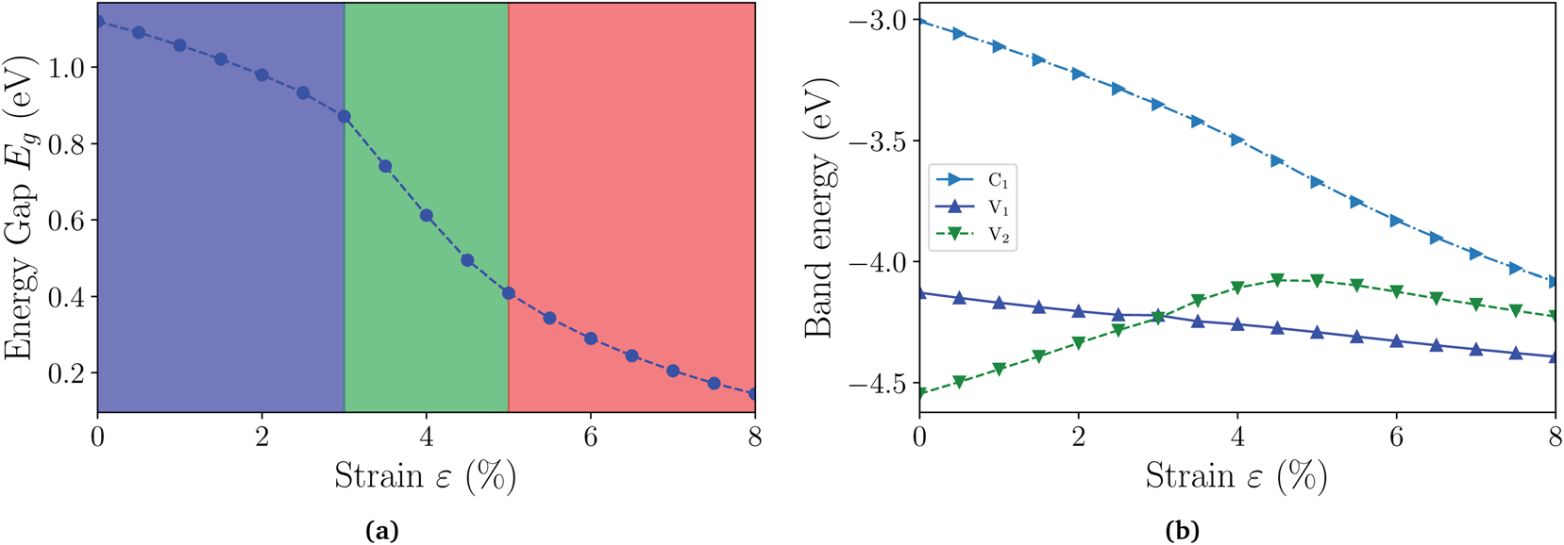
(a) The energy gaps *E*_g_ (PBE) as a function of biaxial strain partitioned into three regions: in region I, the gap is direct at Γ point; in region II, the gap is still direct, but decrease faster; in region III, the gap is indirect. (b) The energies of near-gap energy states C_1_, V_1_, and V_2_ as a function of strain. The band energy values are computed with respect to the *x*–*y* plane average electrostatic potential energy in the vacuum region.

With the fully hydrogenated ZnSb monolayer determined to have a band gap, we then investigate the strain effect on its electronic properties by applying biaxial strains ranging from 0% to 8%. In Fig. 4(a) we plot the variation of band gaps *E*_g_ as a function of the biaxial tensile strain *ε*. Overall the band gap exhibits a monotonic reduction from 1.12 eV at 0% strain to 0.14 eV at 8% strain. A close examination reveals that the band gap variation can be partitioned into three regions based on the variation rate and band gap nature: in region I (0 < *ε* < 3%), the band gap is direct with VBM and CBM at Γ point and the variation rate is −8.27 eV; in region II (3% < *ε* < 5%), the band gap is still direct at Γ, but the variation rate is −23.15 eV; in region III (5% < *ε* < 8%), the band gap is indirect with VBM at a point away from Γ to X and CBM still at Γ and the variation rate is −8.96 eV. We also investigate the electronic structures under biaxial strain using HSE functional (Fig. S4†). The PBE and HSE functionals give qualitatively and even quantitatively similar band gap variations. So the HSE gaps can be regarded as a nearly constant shift of the PBE values, in agreement with previous reports.^47,48^

The computed dependence of band gaps on the strain could be understood from the perspective of bonding nature of VBM and CBM whose energy difference determines the band gap. Two neighboring orbitals can interact to form bonding and anti-bonding energy states that respond to the strain in the opposite manner. The tensile strain leads to an increase in the energy for a bonding state and a reduction in the energy for an anti-bonding state.^49^ We can first focus on the strain-free ZnSb monolayer and plot the partial charge densities for one conduction band state labeled as C_1_ in Fig. 3(e) and two valence band states labeled as V_1_, V_2_ in Fig. 3(f) and (g). We find that the C_1_ state is of anti-bonding character and is mainly contributed by s orbitals of Zn and Sb. The V_1_ is a anti-bonding state mainly composed of Sb-p_*x*_ orbitals while the V_2_ is a bonding state formed between p_*y*_ orbitals of Sb and Zn. Because the band gap variation as demonstrated in Fig. 4(a) is a result of the relative positions of energy states V_1_, V_2_ and C_1_ of different bonding natures, in Fig. 4(b) we show how these energy states respond to the biaxial tensile strain. To find how the VBM and CBM values vary as a function of biaxial strains, a common reference energy in different configurations is needed. For this purpose, we compute the *x*–*y* plane averaged electrostatic potential energy. The zero energy is set to the electrostatic potential energy at the vacuum region, located farthest to the ZnSb sheet. In region I, the energy of V_1_ state is decreasing while the energy of V_2_ state is increasing. As a result, they gradually move towards each other and become degenerate at a strain of 3%. With further increasing strain this trend continues until the VBM moves away from Γ point and the band gap becomes indirect at 5% strain. For the state C_1_, its energy continuously decreases in a linear manner as a function of strain.

### 3.4 Transport anisotropy

**Fig. 5:**
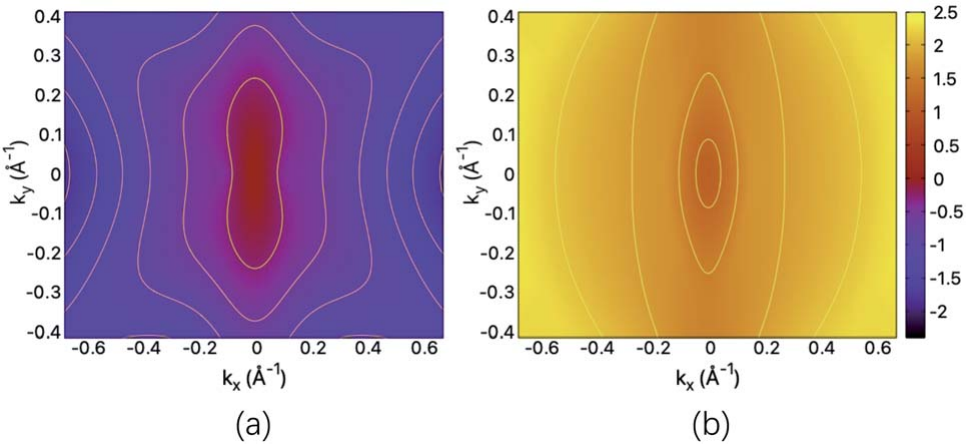
The energy contour of (a) VBM and (b) CBM for the fully hydrogenated ZnSb sheet. The scale bar represents the energies of the electronic states and the Fermi level is set at the zero energy.

The structural and mechanical anisotropy suggests the anisotropy in transport property which can be qualitatively shown by the energy dispersions of near-gap energy states. For the fully hydrogenated ZnSb sheet, in Fig. 5(a) and (b) we plot the energy contour for VBM and CBM, respectively. For VBM, the iso-energy curves show that the energy values decrease more rapidly along *k*_*x*_ direction in comparison with *k*_*y*_ direction. Since the band dispersion determines the curvature and therefore, the effective masses of electron carriers are expected to exhibit orientation anisotropy. For the CBM, we have similar trend along *k*_*x*_ and *k*_*y*_ directions. This is consistent with the electronic structure plotted in Fig. 3(c).

**Fig. 6:**
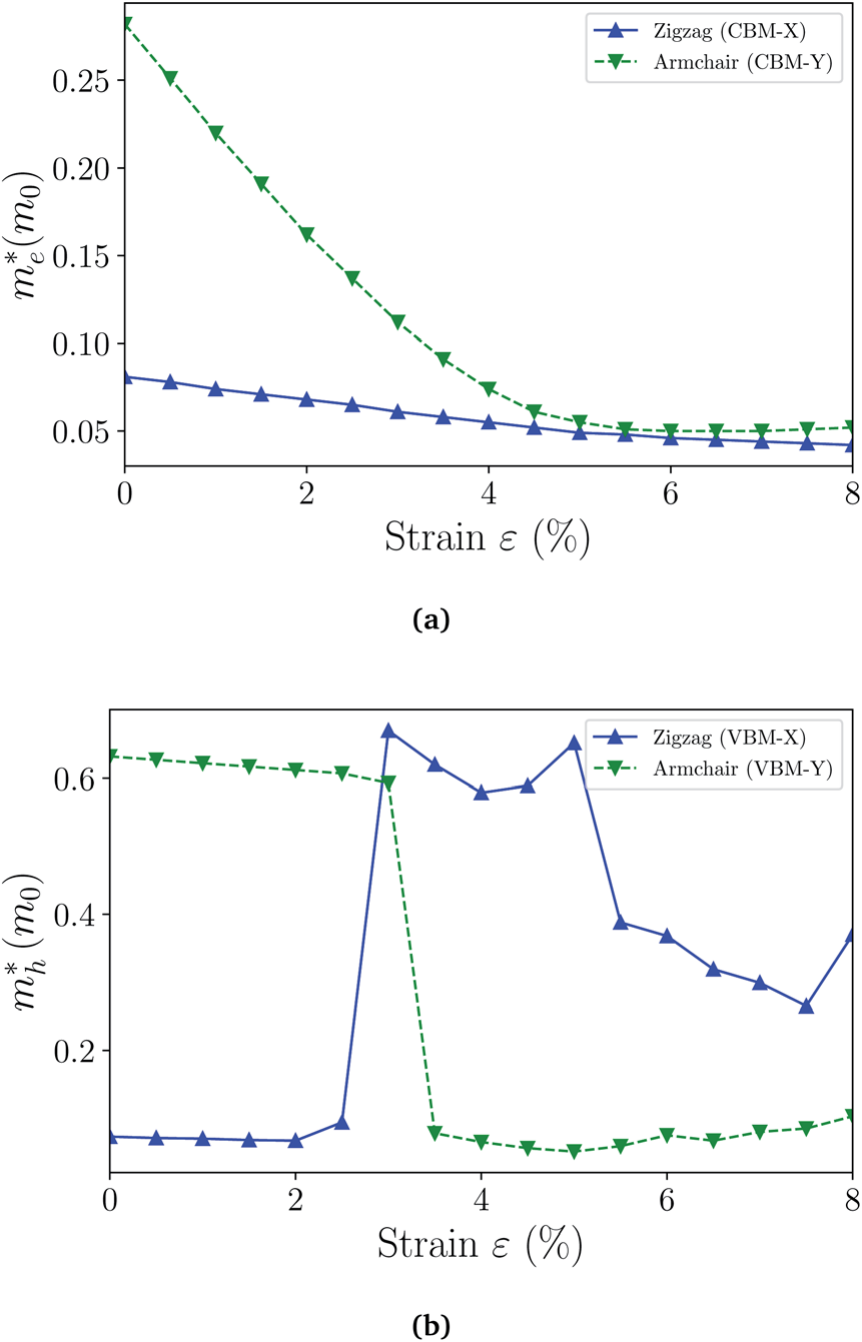
The effective masses of electron (a) and hole (b) as a function of strain. The switching of the preferred transport direction occurring at about 3% strain corresponds to the band crossing demonstrated in Fig. 4(b) and S3.†

Now we quantitatively look into the transport property related to the band dispersion by performing the calculations of the effective masses of charge carriers. The effective mass m* is defined as 
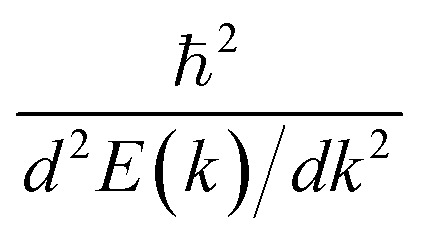
, where ℏ is the reduced Planck constant, *E*(*k*) is the energy band dispersion and *k* is the magnitude of the wave-vector in the momentum space. Therefore, the effective masses of the electrons 
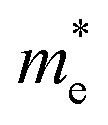
 and holes 
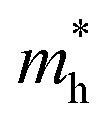
 can be computed *via* parabolic fittings of energy bands near band extremes at Γ point (around Γ point for indirect band gaps). Because of the structure anisotropy, the transport along armchair and zigzag directions have been considered. We present the computed effective masses in Fig. 6. For the fully hydrogenated ZnSb monolayer in a strain-free state, the effective masses of the electron 
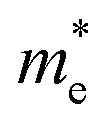
 are calculated to be 0.08 *m*_e_ and 0.28 *m*_e_ in the zigzag and armchair directions, respectively. The effective masses of the hole 
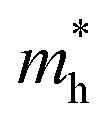
 are predicted to be 0.07 *m*_e_ and 0.63 *m*_e_ in the zigzag and armchair directions, respectively. The relative magnitude indicates that the charge carriers prefer to transport along zigzag direction.

When the biaxial strain is applied, 
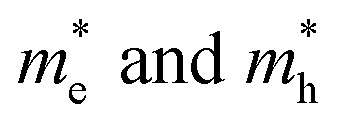
 exhibit much different responses. 
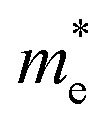
 along armchair direction is slightly reduced while 
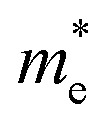
 along zigzag direction is largely modified and decreases to 0.05 *m*_0_. So the biaxial strain reduces the transport anisotropy for electrons. For 
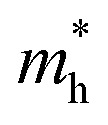
, the strain response is much different. For small strains less than 3%, 
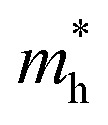
 is not sensitive to the strain and the holes remain to transport along zigzag direction. With further increasing the strain, 
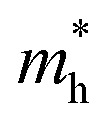
 experiences a jump/drop at about 3% strain, leading to a switching of preferred transport path to be along armchair direction. Later at 5% strain 
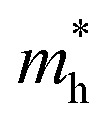
 along zigzag direction drops again but still maintain at a level much higher the 
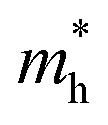
 along armchair direction. Peng *et al.* report an all-electrical conformal five-contact (C5C) method to reveal the in-plane crystal orientation by determining the anisotropic resistivity in exfoliated black phosphorus.^50^ Similarly, the transport anisotropy and its strain tuning in ZnSb sheets indicate that the C5C transport measurement could be utilized to detect the in-plane crystal orientations and to check the existence of strain.

## 4 Summary

In summary, we investigate the physical properties of the hydrogenated two-dimensional ZnSb monolayers with density-functional theory simulations, and demonstrate the strain engineering is an effective routine in tuning the mechanical, electronic and transport properties of fully hydrogenated ZnSb monolayer sheets. We observe a large mechanical anisotropy between armchair and zigzag directions and an effective tuning by applying biaxial tensile strains. We find that with full hydrogenation, the two-dimensional ZnSb monolayer is semiconducting with a direct band gap. During the strain engineering process, the band gap displays a descent trend with the incremental strain and direct-to-indirect transition for larger strain. We understand the band gap dependence on strain by analyzing the bonding nature of near-gap states and their strain response. The transport property exhibits a strong orientation anisotropy and strain tunability, demonstrated by the effective masses of charge carriers along armchair and zigzag directions. Our simulations suggest that hydrogenation and strain engineering could be used to effectively tune the physical properties of novel two-dimensional ZnSb materials and enrich the applications of these newly discovered two-dimensional materials in electronics and opt-electronics.

## Conflicts of interest

There are no conflicts to declare.

## Supplementary Material

RA-012-D1RA08619G-s001
