# Supplementary material for: Direct band gap and anisotropic transport of ZnSb monolayers tuned by hydrogenation and strain
Source: RSC Adv. 2022 Jan 20;12(5):2693–700. doi: 10.1039/d1ra08619g (PMC8979137; doi:10.1039/d1ra08619g)
Supplement: RA-012-D1RA08619G-s001 [file RA-012-D1RA08619G-s001.pdf]

Electronic Supplementary Material (ESI) for RSC Advances.
This journal is © The Royal Society of Chemistry 2022

Supporting information

Direct band gap and anisotropic transport of ZnSb monolayers tuned by hydrogenation and strain

Zhizi Guan[a], Wei Yang[a], Hongfa Wang[a], Hailong Wang[a], and Junwen Li[b]

[a] CAS Key Laboratory of Mechanical Behavior and Design of Materials, Department of Modern Mechanics, CAS Center for Excellence in Complex System Mechanics, University of Science and Technology of China, Hefei, Anhui 230027, China
E-mail: hailwang@ustc.edu.cn

[b] DFTWorks LLC, Oakton, VA 22124, USA
E-mail: junwen.li@dftworks.com

To check the mechanical stability of hydrogenated ZnSb monolayers, we carry out the calculations of elastic stiffness constants of ZnSb monolayers with full hydrogenation. With the Born-Huang stability criteria as described by

$$C_{11} > 0,\ C_{22} > 0,\ C_{11} > |C_{12}|,\ C_{66} > 0,$$

the fully hydrogenated 2D ZnSb monolayers under strains of 0% and 8% are found to be mechanically stable as shown in table S1.

Table S1. Elastic Stiffness Constants of fully hydrogenated 2D ZnSb monolayer.

**Strain**	**C_{11}(N/m)**	**C_{12}(N/m)**	**C_{22}(N/m)**	**C_{66}(N/m)**
0%	44.000	7.119	31.255	14.554
8%	21.539	4.381	31.027	11.408

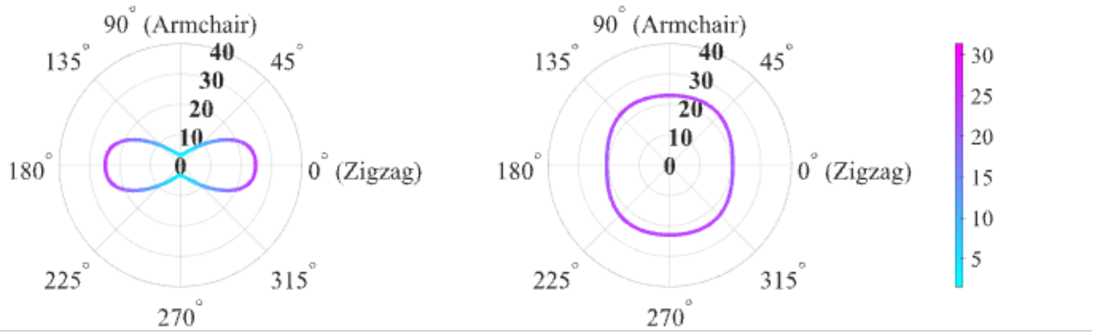

(a) (b)

Figure S1. Young's Modulus of ZnSb monolayer sheet as a function of the in-plane angle θ with θ corresponding to the a axis (zigzag) under (a) 3% and (b) 5.5% strain.

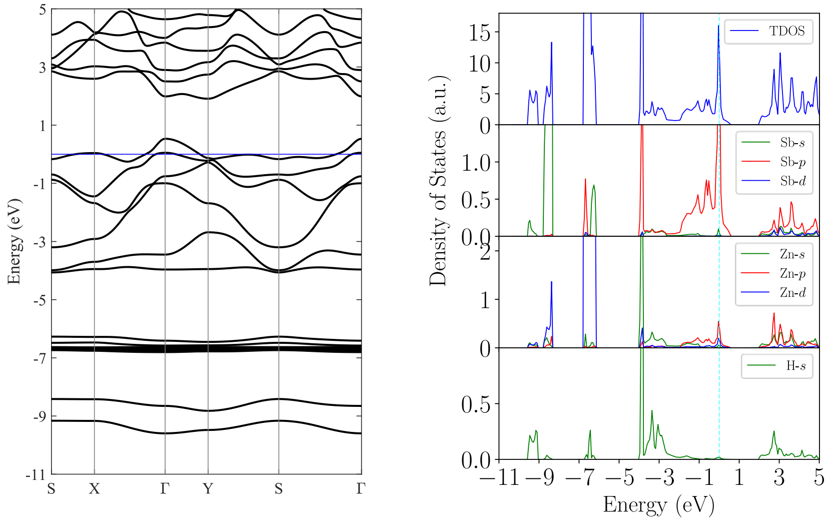

(a) (b)

Figure S2. (a) Electronic band structure (PBE) and (b) total and orbital-projected density of states for the half hydrogenated ZnSb monolayer sheet. The Fermi level is set to zero.

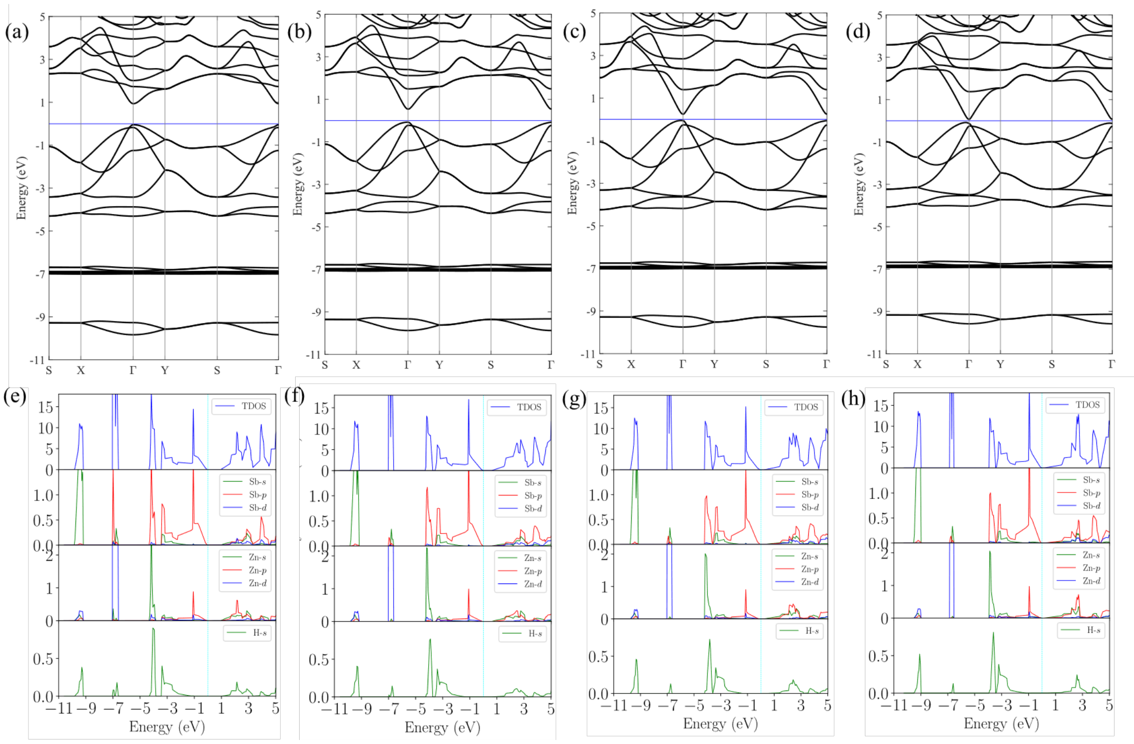

Figure S3. (a)-(d) Calculated electronic band structures (PBE) and (e)-(h) total and orbital-projected density of states for the fully hydrogenated ZnSb monolayer under strains of 2%, 4%, 6%, and 8%, respectively.

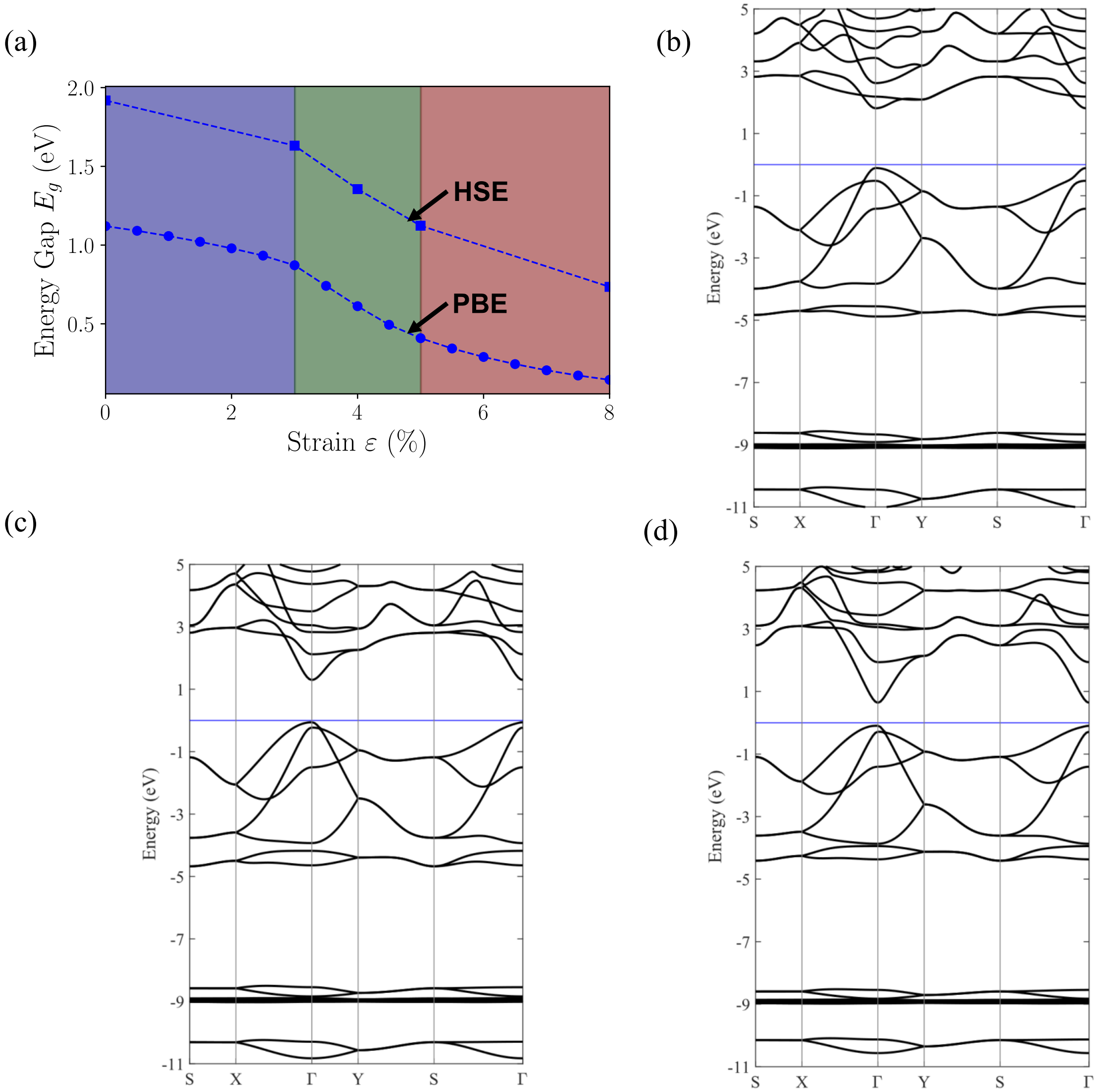

Figure S4. (a) The energy gaps E_g as a function of biaxial strain using PBE and HSE functionals. (b)-(d) Calculated electronic band structures (HSE) for the fully hydrogenated ZnSb monolayer sheet under strains of 0%, 4% and 8%, respectively. The Fermi level is set to zero.

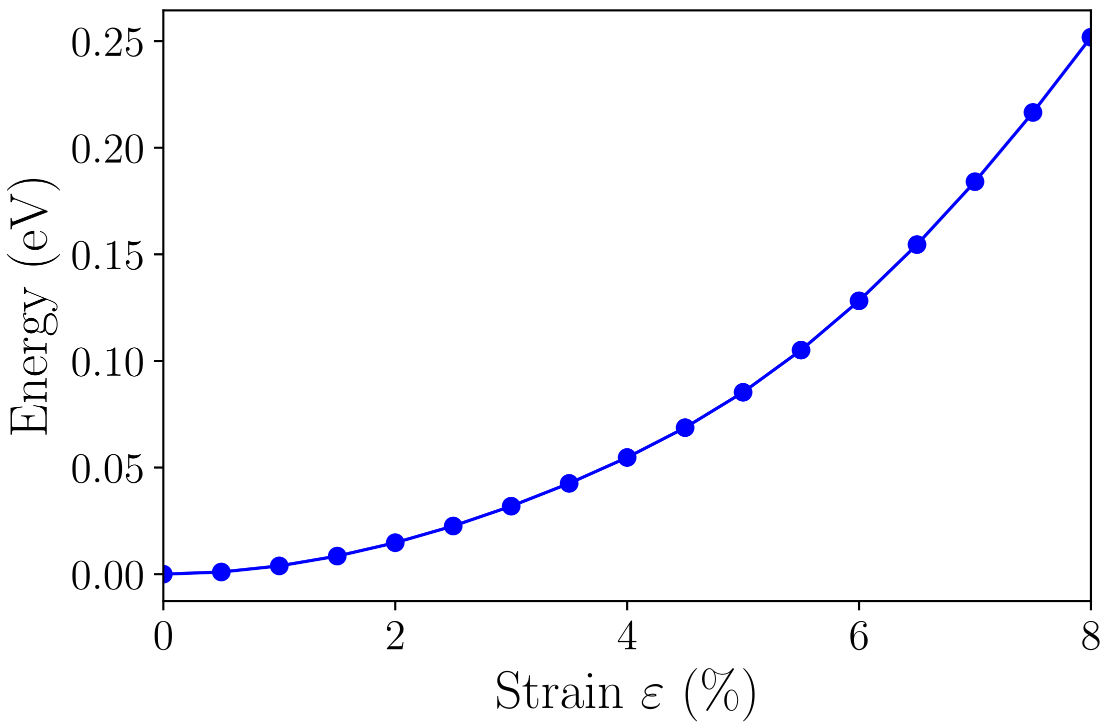

Figure S5. Total energy of the fully hydrogenated sheet as a function of strain. The total energy of the unstrained sheet is set to zero.
